# Use of an Automated Quantitative Analysis of Hippocampal Volume, Signal, and Glucose Metabolism to Detect Hippocampal Sclerosis

**DOI:** 10.3389/fneur.2018.00820

**Published:** 2018-10-04

**Authors:** Wen-han Hu, Li-na Liu, Bao-tian Zhao, Xiu Wang, Chao Zhang, Xiao-qiu Shao, Kai Zhang, Yan-Shan Ma, Lin Ai, Jun-ju Li, Jian-guo Zhang

**Affiliations:** ^1^Beijing Neurosurgical Institute, Beijing Tiantan Hospital, Capital Medical University, Beijing, China; ^2^Department of Pathology, Beijing Fengtai Hospital, Beijing, China; ^3^Department of Neurosurgery, Beijing Tiantan Hospital, Capital Medical University, Beijing, China; ^4^Department of Neurology, Beijing Tiantan Hospital, Capital Medical University, Beijing, China; ^5^Department of Neurosurgery, Beijing Fengtai Hospital, Beijing, China; ^6^Nuclear Medicine, Beijing Tiantan Hospital, Capital Medical University, Beijing, China; ^7^Department of Neurosurgery, Hainan General Hospital, Haikou, China

**Keywords:** mesial temporal lobe epilepsy, hippocampal sclerosis, MRI, ^18^FDG-PET, quantitative analysis

## Abstract

**Purpose:** Magnetic resonance imaging (MRI) and positron emission tomography (PET) with ^18^F-fluorodeoxyglucose (^18^FDG) are valuable tools for evaluating hippocampal sclerosis (HS); however, bias may arise during visual analyses. The aim of this study was to evaluate and compare MRI and PET post-processing techniques, automated quantitative hippocampal volume (Q-volume), and fluid-attenuated inversion-recovery (FLAIR) signal (Q-FLAIR) and glucose metabolism (Q-PET) analyses in patients with HS.

**Methods:** We collected MRI and ^18^FDG-PET images from 54 patients with HS and 22 healthy controls and independently performed conventional visual analyses (CVA) of PET (CVA-PET) and MRI (CVA-MRI) images. During the subsequent quantitative analyses, the hippocampus was segmented from the 3D T1 image, and the mean volumetric, FLAIR intensity and standardized uptake value ratio (SUVR) values of the left and right hippocampus were assessed in each subject. Threshold confidence levels calculated from the mean volumetric, FLAIR intensity and SUVR values of the controls were used to identify healthy subjects or subjects with HS. The performance of the three methods was assessed using receiver operating characteristic (ROC) curves, and the detection rates of CVA-MRI, CVA-PET, Q-volume, Q-FLAIR, and Q-PET were statistically compared.

**Results:** The areas under the curves (AUCs) for the Q-volume, Q-FLAIR, and Q-PET ROC analyses were 0.88, 0.41, and 0.98, which suggested a diagnostic method with moderate, poor, and high accuracy, respectively. Although Q-PET had the highest detection rate among the two CVA methods and three quantitative methods, the difference between Q-volume and Q-PET did not reach statistical significance. Regarding the HS subtypes, CVA-MRI, CVA-PET, Q-volume, and Q-PET had similar detection rates for type 1 HS, and Q-PET was the most sensitive method for detecting types 2 and 3 HS.

**Conclusions:** In MRI or ^18^FDG-PET images that have been visually assessed by experts, the quantification of hippocampal volume or glucose uptake can increase the detection of HS and appear to be additional valuable diagnostic tools for evaluating patients with epilepsy who are suspected of having HS.

## Introduction

Mesial temporal lobe epilepsy (MTLE) is the most frequent form of partial drug-resistant epilepsy in adults, and hippocampal sclerosis (HS) is the main pathological substrate, accounting for 17–44.5% of surgical candidates in epilepsy centers (1–3). The identification of HS is clinically important, as these patients have a 68% chance of becoming seizure free after surgery (4). In addition to semiological and electrophysiological studies, neuroimaging modalities, including magnetic resonance imaging (MRI) and positron emission tomography (PET) with ^18^F-fluorodeoxyglucose (^18^F-FDG), provide useful information for the diagnosis of HS.

A typical HS MRI is characterized by a reduced hippocampal volume, enlargement of Ammon's horn and hyperintensity of hippocampal structures in T2-weighted and fluid-attenuated inversion-recovery (FLAIR) sequences (5, 6). Interictal PET imaging is widely used in the presurgical evaluation of patients with MTLE in many epilepsy centers, as temporal glucose hypometabolism has been reported to predict favorable seizure outcomes after surgery (7). However, in clinical practice, a conventional MRI or PET analysis is a visual and qualitative method. The ability to detect structural or metabolic changes in the hippocampus strongly depends on both the quality of the imaging data and the training and experience of the interpreting rater. The hippocampal volume, signal, and glucose uptake values are difficult to compare bilaterally in an asymmetric scan, and mild changes tend to be overlooked by image readers who are blinded to the clinical manifestations or electroencephalography (EEG) data.

Quantitative analyses of the corresponding data from the whole hippocampus may avoid the abovementioned limitations of visual analyses. MRI post-processing techniques, including quantification of the hippocampal volume, signal and shape, have been developed to improve the detection of HS (8–11). Several studies have quantitatively analyzed the asymmetry of hippocampal volume, signal and glucose uptake in patients with HS (12, 13); however, bilateral HS with symmetrical abnormities might be overlooked due to a lack of comparison with normal control subjects. In the present study, we compared the MRI and PET data from individual patients with HS with data from healthy controls to determine the lateralizing values of quantitative analyses of hippocampal volume (Q-volume), signal (Q-FLAIR) and glucose metabolism (Q-PET). We also explored whether the sensitivity of each modality differed in patients with different HS subtypes, as classified by the International League Against Epilepsy (ILAE) grading system (14).

## Materials and methods

### Patients and controls

We included patients who were consecutively enrolled from January 2015 to December 2017 at the Beijing Tiantan-Fengtai Epilepsy Center using the following protocols: (1) an anterior temporal lobectomy or selective amygdalohippocampectomy was performed; (2) the histopathological finding was HS, according to ILAE diagnostic methods (14); (3) the patient was seizure-free at the time of the last follow-up (8–35 months, mean 19.78 ± 7.00 months; and (4) presurgical ^18^FDG-PET and 3D T1 images were available. Twenty-two healthy subjects with similar ages to the included patients with HS were also recruited in this study. The healthy subjects were free from neurological or psychiatric disorders, and their cerebral MRI scans were normal. All patients and control volunteers underwent high-resolution MRI and ^18^FDG-PET scans using the protocols described below. MRI scans were performed on a 3.0-T Siemens Verio scanner (Siemens Medical Systems, South Iselin, NJ), including 3D T1 sagittal magnetization-prepared rapid gradient echo (MPRAGE) (TR/TE 1900/2.53, TI 900, matrix 256 × 256, 1.0 mm thickness), T2 axial (TR/TE 7030/110, matrix 256 × 320, 3 mm thickness), FLAIR axial (TR/TE 8000/94, TI 2371.5, matrix 424 × 512, 3 mm thickness), FLAIR sagittal (TR/TE 8000/96, TI 2371.2, matrix 236 × 256, 3 mm thickness), and FLAIR coronal (TR/TE 8000/96, TI 2371.2, matrix 408 × 512, 3 mm thickness) sequences. PET scans were obtained in the interictal state with ^18^FDG under standard resting conditions (eyes closed in dimmed ambient light). The ^18^FDG-PET examination was performed using a GE Discovery ST PET-CT system (General Electric Medical Systems, Milwaukee, WI, USA) (300 mm FOV, matrix 192 × 192, and 3.27 mm slice thickness). An IV injection of ^18^FDG at a mean dose of 220 MBq/70 kg body weight was administered. Reconstructed images were corrected for attenuation using transmission scans obtained from a germanium source. No patients had an ictal event <6 h before or during the PET scan. Other clinical information was extracted from the medical records, including gender, age at seizure onset, epilepsy duration, seizure frequency, semiology, and scalp-EEG findings.

### Conventional visual analysis (CVA) of MRI and FDG-PET images

The MRI and FDG-PET images from the patients and controls were mixed and independently analyzed by two epilepsy (Xiao-qiu Shao) and neuroimaging (Lin Ai) experts who were blinded to the subjects' characteristics, and discrepancies were resolved through discussion. PET hypometabolism was qualitatively defined as decreased FDG uptake in the temporal lobe.

### MRI post-processing

**Figure 1:**
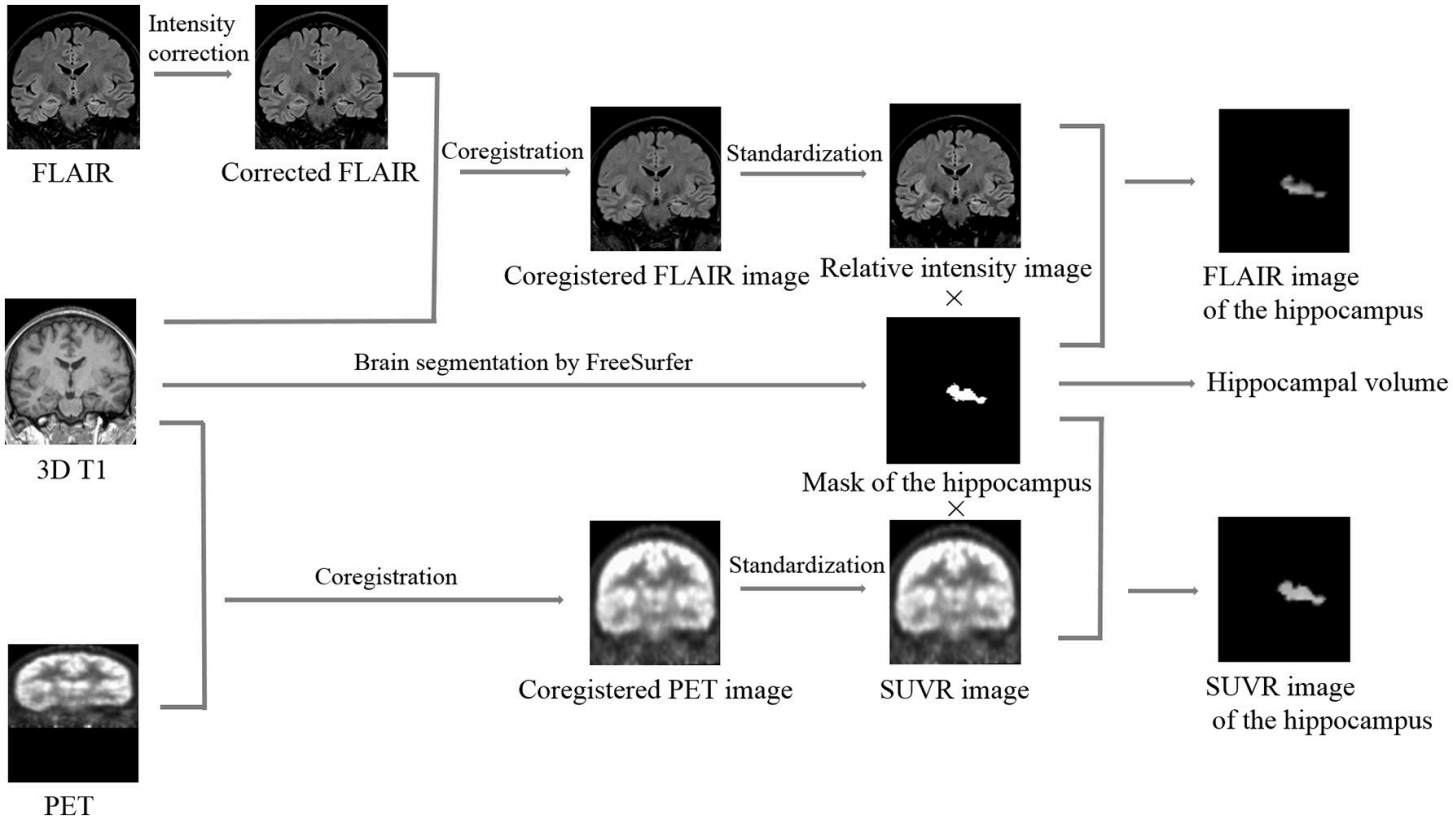
Summary of the image processing steps required for quantifying the hippocampal volume, relative FLAIR intensity, and relative glucose uptake. Brain segmentation: different cerebral structures, including the hippocampus, were automatically segmented by FreeSurfer, and the mask images and the volumetric values of the left and right hippocampus were calculated. Intensity correction: potential intensity inhomogeneities in the coronal FLAIR image were removed using the “segment” algorithm in SPM8. Coregistration: the corrected FLAIR or FDG-PET image was coregistered and resliced to the 3D T1 image using the default parameters of SPM8. Standardization: the coregistered FLAIR or FDG-PET image was divided by the mean intensity value of its corresponding skull-stripped image voxel by voxel to obtain an image of the relative FLAIR intensity or SUVR, respectively. Calculation of the hippocampal FLAIR or SUVR image: the relative FLAIR intensity or SUVR image was multiplied by the mask image of the hippocampus to obtain the FLAIR or SUVR image of the hippocampus, respectively. SUVR, standardized uptake value ratio.

Automated hippocampal segmentation and volumetric analysis were performed with FreeSurfer software (Martinos Center for Biomedical Imaging, Harvard-MIT, Boston, USA; https://surfer.nmr.mgh.harvard.edu) using a MPRAGE sequence, and mask files of the hippocampus and the whole brain were created after segmentation (Figure 1). Hippocampal volumes were standardized by dividing them by the individual supratentorial volume. The workflow of the quantitative FLAIR intensity analysis was similar to the method proposed by Huppertz et al. (9). Post-processing was exclusively performed with SPM8 (statistical parametric mapping software, Wellcome Trust Centre for Neuroimaging, London, UK; http://www.fil.ion.ucl.ac.uk/spm). The six steps of FLAIR image processing were fully automated using a MATLAB® script (Figure 1). In Step 1, the function of bias correction in the “segmentation” algorithm was used to correct the intensity bias of individual coronal FLAIR images; in Step 2, the corrected FLAIR image was coregistered and resliced to the MPRAGE image using the default parameters of SPM8; In Step 3, the brain was extracted from the FLAIR image by multiplying the brain mask file and calculating the mean intensity of the skull-stripped FLAIR image; in Step 4, the coregistered FLAIR image was divided by the mean intensity value measured in Step 3 voxel by voxel to obtain a standardized FLAIR image; In Step 5, the standardized FLAIR image was multiplied by the left and right hippocampal mask images to calculate corresponding hippocampal FLAIR images, and in Step 6, the mean intensities of left and right hippocampal FLAIR images were calculated.

### FDG-PET image post-processing

Similar to FLAIR image post-processing procedures, the whole workflow of FDG-PET image post-processing included 5 steps (Figure 1): Step 1, coregistration and reslicing; Step 2, calculating the mean intensity of the skull-stripped FDG-PET image; Step 3, deriving an image of the standardized uptake value ratio (SUVR) from the coregistered FDG-PET image by dividing by the mean intensity value measured in step 2 voxel by voxel; Step 4, calculating hippocampal SUVR images; and Step 5, calculating the mean intensities of left and right hippocampal SUVR images.

### Data analysis

The results for hippocampal volume, FLAIR signal and SUVR obtained from patients and controls are shown in a scatter plot, with the x- and y-axis representing the mean volumetric, FLAIR intensity and SUVR values of the right and left hippocampus, respectively. An error ellipse with a manually set confidence level (CL) was calculated from the mean volumetric, FLAIR intensity and SUVR values of the controls using the error_ellipse.m program written by A. J. Johnson and obtained from the MATLAB® central file exchange (http://www.mathworks.com/matlabcentral/fileexchange/4705-error-ellipse). The sensitivity and specificity of each method were assessed by calculating the proportion of patients with HS outside the ellipse who displayed correct lateralization and the proportion of controls within the ellipse, respectively. We varied the area of the error ellipse by changing the value of CL (from 1 to 99%) to evaluate the sensitivity and specificity of each quantitative method. Youden's index (Youden's index = sensitivity + specificity – 1) was calculated to obtain the optimal value of CL. Receiver-operating characteristic (ROC) curves and the area under the ROC curve (AUC) were used to assess the feasibility of using Q-volume, Q-FLAIR and Q-PET to detect HS.

### Statistical analysis

ROC curves were generated using GraphPad Prism 7.0 software (GraphPad Software Inc., San Diego, California, USA). The differences in detection rates between each method were compared using Fisher's exact test (two-tailed). Significance was defined as *P* < 0.05. Statistical tests were performed using SPSS 22.0 (IBM Inc., New York, USA).

## Results

### General clinical data

Fifty-four patients (33 females and 21 males) were included, and the mean age at surgery was 26.37 ± 7.82 years. Among the 54 patients, 20 patients underwent stereoelectroencephalography (SEEG) monitoring before surgery, and 36 patients underwent left resection. The histopathological studies revealed that 33, 17 and 4 specimens were type 1, type 2 and type 3 HS, respectively. General clinical characteristics, video-EEG findings, neuroimaging findings, and surgical outcomes of the 54 patients are provided in Table 1. No significant differences in the distribution of gender (*p* = 0.52) or age (*p* = 0.13) were observed between the patients and controls.

**Table 1:** Clinical characteristics, neuroimaging findings, and surgical outcomes of 54 patients.

**Patient**	**FC**	**Interictal EEG**	**Ictal EEG**	**Resection side**	**CVA-MRI**	**CVA-PET**	**Q-volume**	**Q-FLAIR**	**Q-PET**	**SEEG**	**HS type**	**Outcome**	**Follow-up (mon)**
1	YES	Non-lat	RT > LT	R	n	w	p	p	p	Yes	2	1a	30
2	NO	LT	LFT	L	n	p	n	n	p	Yes	3	1a	27
3	NO	LFT	LT	L	n	n	w	w	n	Yes	3	1a	27
4	YES	Non-lat	Non-lat	L	p	p	p	w	p	Yes	2	1a	21
5	NO	LFT	LT	L	p	p	p	p	p	Yes	1	1a	19
6	YES	LT	LF	L	n	n	p	p	p	Yes	3	1a	18
7	NO	LT	Non-lat	L	p	p	p	p	p	Yes	1	1b	28
8	NO	RFT	RT	R	p	p	p	p	p	No	2	1d	30
9	NO	LFT	LH	L	n	p	w	n	p	Yes	2	1a	34
10	NO	LT	LH	L	p	n	p	p	p	No	2	1a	25
11	NO	LT	LT	L	p	p	p	p	p	No	1	1b	24
12	YES	RT	RT	R	p	p	p	p	p	No	2	1a	17
13	NO	LH>RH	LT	L	n	n	p	n	p	No	2	1a	17
14	NO	LT	LFT	L	n	p	p	p	p	Yes	2	1a	17
15	YES	Non-lat	RT	R	p	n	p	p	p	No	1	1a	25
16	YES	LFT > RFT	LT	L	p	p	p	p	p	No	1	1a	18
17	NO	RT	RFT	R	p	p	p	n	p	Yes	1	1a	34
18	YES	RT	RFT	R	p	p	p	n	p	No	1	1a	20
19	NO	RT > LT	RFT	R	p	p	p	n	p	No	1	1d	29
20	NO	LFT	LFT	L	p	p	p	p	p	Yes	2	1a	23
21	YES	LFT	LT	L	p	n	p	n	p	Yes	1	1a	15
22	NO	Non-lat	LH	L	n	n	w	n	p	Yes	2	1a	25
23	NO	Non-lat	LT	L	p	p	p	p	p	No	2	1a	26
24	NO	LT	LT	L	p	p	p	w	p	No	1	1a	20
25	YES	Non-lat	RT	R	p	p	p	p	p	No	3	1d	23
26	YES	LT	LT	L	p	p	p	n	p	No	1	1a	20
27	NO	LFT	LT	L	p	p	p	w	p	No	1	1a	28
28	YES	Non-lat	LT	L	p	p	p	n	p	No	2	1a	22
29	YES	LFT	LFT	L	p	p	p	n	p	No	2	1a	19
30	NO	RT	RT	R	p	p	p	p	p	No	1	1a	15
31	NO	LFT	LFT	L	p	p	p	w	p	No	1	1d	27
32	YES	LFT	LT	L	p	p	p	n	p	No	1	1a	31
33	YES	Non-lat	Non-lat	R	n	n	p	p	p	Yes	2	1a	13
34	NO	Non-lat	Non-lat	R	p	p	p	n	p	No	1	1a	12
35	NO	LT	LT>RT	L	p	p	p	w	p	No	1	1a	12
36	NO	RFT > LFT	RFT	R	p	n	p	p	p	No	1	1a	13
37	YES	Non-lat	RFT	R	p	p	p	p	p	No	1	1a	13
38	NO	LT	LT	L	p	p	p	n	p	No	1	1a	12
39	YES	LFT	LFT	L	p	p	p	w	p	No	1	1a	35
40	YES	LFT	LH	L	p	p	p	n	p	No	1	1d	15
41	YES	Non	Non	L	p	p	p	w	p	Yes	1	1b	13
42	YES	LT	LFT	L	p	p	p	n	p	No	1	1a	17
43	YES	LFT	LFT	L	n	p	p	w	p	No	2	1b	13
44	YES	RT	RT	R	p	p	p	p	p	No	1	1a	12
45	YES	RFT > LFT	RH>LH	R	n	p	p	w	p	Yes	2	1a	17
46	NO	LT	LT	L	p	p	p	w	p	No	1	1a	8
47	NO	LT	LT	L	p	p	p	w	p	No	1	1a	15
48	YES	LT	LFT	L	p	p	p	n	p	Yes	1	1a	13
49	YES	RT	RF	R	p	p	p	n	p	Yes	1	1d	16
50	NO	LFT	LFT	L	p	p	p	n	p	No	1	1b	14
51	NO	LT	LT	L	p	p	p	p	p	No	1	1a	17
52	YES	LFT	LH	L	p	p	p	n	p	No	2	1a	8
53	YES	RFT	RT	R	p	p	p	p	p	Yes	1	1a	12
54	NO	RT	RH	R	p	p	p	n	p	Yes	1	1a	14

*CVA, conventional visual analysis; F, frontal; FC, febrile convulsion; H, hemispheric; HS, hippocampal sclerosis; L, left; n, negative; non-lat, non-lateralized; p, positive; R, right; SEEG, stereoelectroencephalography; T, temporal; w, wrong lateralization*.

### Performance of CVA-MRI and CVA-PET

Structural abnormalities in MRI images were detected by CVA in 43 patients, and all detected MRI abnormalities had lateral concordance with EEG findings; thus, the sensitivity of CVA-MRI was 79.63%. Regarding the HS subtype, 100, 52.94, and 25% of type 1, type 2, and type 3 cases were detected, respectively. The FDG-PET visual analysis detected 45 patients with temporal hypometabolism (14 right and 31 left). The remaining 9 patients were considered normal in FDG-PET images based on visual analyses. Among the 45 patients with temporal hypometabolism determined by a visual analysis, 44 patients had side concordances between FDG-PET images and EEG findings. The remaining patient exhibited left temporal hypometabolism in the visual analysis, which was contralateral to the epileptic focus (as defined by scalp EEG and confirmed by SEEG). As mentioned above, the overall sensitivity of CVA-PET was 81.48%, and the sensitivity of detecting type 1, type 2, and type 3 HS was 90.91, 70.59, and 50%, respectively. Regarding the controls, 19 and 20 controls were identified as normal by CVA-MRI and CVA-PET, respectively. These values corresponded to a specificity of 86.36 and 90.9%, respectively.

### Performance of quantitative analyses

**Figure 2:**
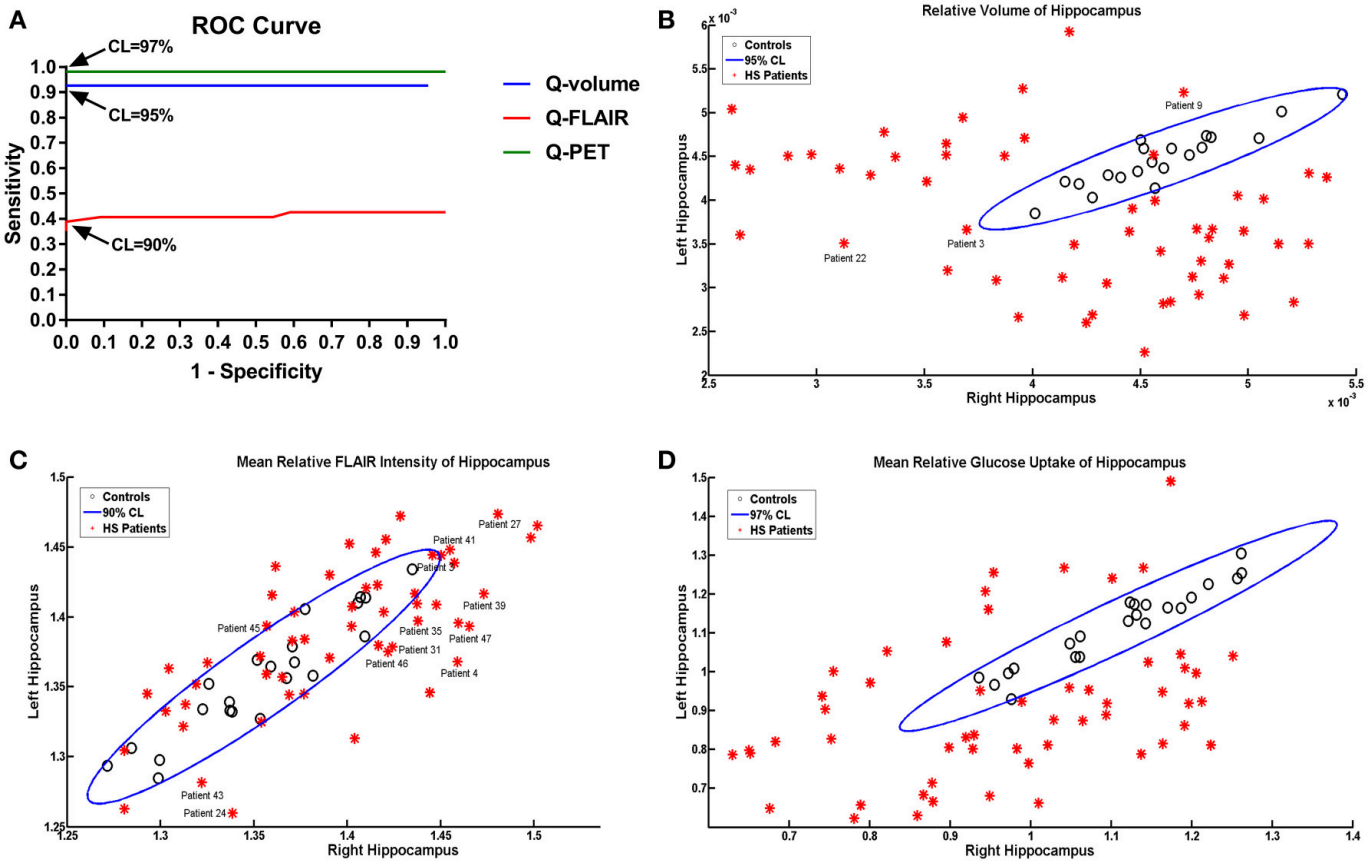
ROC curves showing effects of different confidence level thresholds on sensitivity and specificity **(A)**. Scatter plot displaying the mean volumetric **(B)**, FLAIR intensity **(C)**, and SUVR **(D)** values of the left and right hippocampus in patients with HS and controls. The oblique ellipses represent the 95, 90, and 97% confidence areas determined by controls in Q-volume, Q-FLAIR, and Q-PET analyses, respectively. Please note that patients 3, 9, and 22 were diagnosed as HS with wrong lateralization based on the Q-volume analysis; patients 3, 4, 24, 27, 31, 35, 39, 41, 43, 45, 46, and 47 were diagnosed as HS with wrong lateralization based on the Q-FLAIR anlysis. HS, hippocampal sclerosis; Q, quantitative; ROC, receiver-operating characteristic; SUVR, standardized uptake value ratio.

The ROC analysis showed optimal performance at a CL threshold of 95%, 90% and 97% for Q-volume, Q-FLAIR and Q-PET analysis, respectively (Figure 2A), and the sensitivity of each analysis was 92.59%, 38.89%, and 98.15%, respectively, and the specificity was 100% for each analysis (Figures 2B–D). The areas under the curves for the Q-volume, Q-FLAIR and Q-PET ROC analysis were 0.88, 0.41, and 0.98, which suggested a diagnostic method with moderate, poor, and high accuracy, respectively (Figure 2A). The detection rate of Q-volume, Q-FLAIR, and Q-PET for type 1, type 2 and type 3 HS are summarized in Table 2.

**Table 2:** Detection rates of HS and its subtypes using different modalities.

	**All HS (*n* = 54)**	**Type 1 HS (*n* = 33)**	**Type 2 HS (*n* = 17)**	**Type 3 HS (*n* = 4)**
CVA-MRI	43 (79.63%)	33 (100%)	9 (52.94%)	1 (25%)
CVA-PET	44 (81.48%)	30 (90.91%)	12 (70.59%)	2 (50%)
Q-volume	50 (92.59%)	33 (100%)	15 (88.24%)	2 (50%)
Q-FLAIR	21 (38.89%)	11 (33.33%)	8 (47.06%)	2 (50%)
Q-PET	53 (98.15%)	33 (100%)	17 (100%)	3 (75%)

*CVA, conventional visual analysis; HS, hippocampal sclerosis; Q, quantitative*.

### Comparison of CVA and quantitative methods

**Figure 3:**
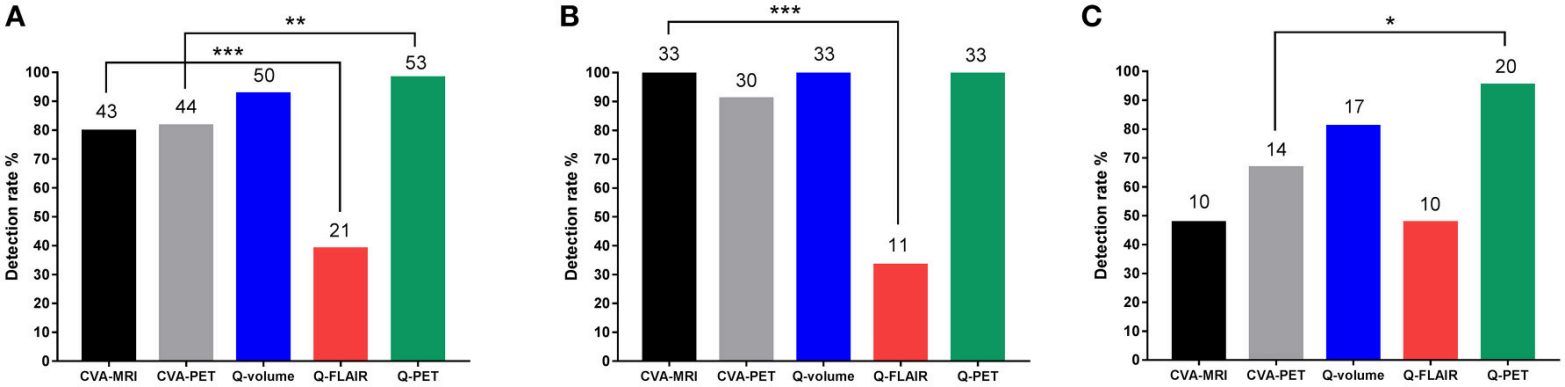
Comparison of detection rates of all types **(A)**, type 1 **(B)**, and types 2 and 3 **(C)** HS using the different modalities. ^*^p < 0.05; ^**^p < 0.01; ^***^p < 0.001. CVA, conventional visual analysis; HS, hippocampal sclerosis; Q, quantitative.

When comparing the detection rates of the two CVA methods and the three quantitative methods, although Q-PET exhibited the highest detection rate, the difference between Q-volume and Q-PET did not reach statistical significance (Q-PET vs. CVA-MRI, *p* = 0.004; Q-PET vs. CVA-PET, *p* = 0.008; Q-PET vs. Q-volume, *p* = 0.363; Q-PET vs. Q-FLAIR, *p* < 0.001) (Table 2 and Figure 3A). We divided patients into multiple-subfield sclerosis (type 1 HS) and single-subfield sclerosis (types 2 and 3 HS) groups. CVA-MRI, CVA-PET, Q-volume, and Q-PET had similar detection rates for type 1 HS (*p* = 0.058) (Table 2 and Figure 3B), and Q-PET was the most sensitive method for detecting types 2 and 3 HS (Q-PET vs. CVA-MRI, *p* = 0.001; Q-PET vs. CVA-PET, *p* = 0.045; Q-PET vs. Q-volume, *p* = 0.343; Q-PET vs. Q-FLAIR, *p* = 0.001) (Figure 3C).

Regarding the controls, the differences in the specificities of the 5 methods did not reach statistical significance (*p* = 0.086).

## Discussion

Not surprisingly, quantitative methods were more sensitive than CVA methods. However, in contrast to our expectations, the mean FLAIR intensity of hippocampus was a parameter with poor accuracy in detecting HS in the present study, although it is routinely used in visual assessments. However, a reduced volume was a reliable and sensitive marker of HS. Because a rater is unable to separately analyze hippocampal volume and FLAIR intensity in a visual assessment, we postulate that this lack of separation is the explanation for the continued effectiveness of visual analyses in clinical practice. Our method of analyzing FLAIR intensity was similar to the method proposed by Huppertz et al., but the results were not consistent. We speculate that the probable explanation for this discrepancy is differences in the inclusion criteria. Instead of a histopathological diagnosis, the majority of patients in the study by Huppertz et al. were diagnosed based on a neuroimaging analysis, which might lead to selection bias because patients with subtle hippocampal changes tend to be overlooked and excluded (9). According to a previous study, reliable visual detection occurs at hippocampal volume ratios <0.7 (15), and the present study also found that a visual inspection only detected 48% of types 2 and 3 HS cases.

The quantitative methods used in the present study had some advantages over manual visual evaluations. First, in contrast to a visual comparison of bilateral hippocampi, the automated method compares the data from individual patients to the data from healthy controls. Therefore, bilateral hippocampal atrophy, which tends to be overlooked in visual analyses, should be easily detected by our method. Bilateral HS cases were not presented in this article because the determination of a pathological diagnosis was impossible in these patients. Second, our method measured global structural or metabolic changes in the hippocampus, while limited sampling during manual analysis may lead to bias, which is associated with an incorrect diagnosis. Third, because the quantitative analysis was an objective method independent of the rater's experiences, the performance might be more stable, and the results might be more reproducible. Fourth, the quantitative analysis improved the sensitivity of detecting subtle structural or metabolic changes. In the present study, quantitative MRI and PET analysis increased the detection rates of types 2 and 3 HS by 33.33 and 28.57%, respectively, compared to a visual assessment. As shown in the study by Coan et al., the quantification of hippocampal volumes and signals in MRIs that are visually inspected by experts increases the detection of HS in 28% of patients with MTLE (16). Finally, the asymmetry of MRI or PET images due to oblique scanning could lead to bias during visual inspections, which did not occur during the quantitative analysis.

Our study also highlighted the relationship between the classification of HS and the detection rates of visual or quantitative analysis. The ILAE proposed a classification of HS in 2013: type 1 refers always to severe neuronal cell loss and gliosis, predominantly in CA1 and CA4 regions, compared to CA1 (HS type 2)- or CA4 (HS type 3)-predominant neuronal cell loss and gliosis (14). With respect to MRI, the degree of volumetric reduction correlates with the severity of neuronal loss within hippocampal subfields. Prior to the ILAE classification, Wyler et al. proposed a semi-quantitative grading system to characterize the severity of HS based on neuronal loss (17), and a subsequent study indicated that the volumetric reduction measured by MRI also correlated well with this pathological grading system (18). In the present study, a visual assessment of MRI identified all type 1 HS cases, while only 50% of types 2 and 3 HS cases were identified using this method. Thus, severe hippocampal atrophy in multiple subfields was obvious and able to be visually detected; therefore, the quantitative analysis is not necessary for these cases. However, atrophy in a single subfield is too subtle or mild to be detected visually, and the quantitative analysis therefore showed its advantages.

Based on our results, Q-PET was more sensitive than Q-volume, although the difference was not statistically significant. The sensitivity of FDG-PET in detecting epileptogenic lesions has been well-recognized not only for MTLE cases but also for neocortical epilepsy cases (13, 19). However, we want to emphasize that FDG-PET is a functional imaging modality that characterizes the different parts of the brain according to metabolic activity. FDG-PET only reflects the metabolic abnormalities in the seizure network and does not represent the essence of a seizure, which is the hyperexcitability of the neurons of the cerebral cortex. Hypometabolic regions are often more broadly distributed than the extent of the seizure onset zone (20), indicating that the hypometabolic region does not precisely represent the seizure onset zone. For example, ipsilateral hippocampal hypometabolism has also been observed in patients with frontal lobe epilepsy, particularly in the anterior cingulate cortex, or orbitofrontal epilepsy. Therefore, in clinical practice, the interpretation of the results of PET images from patients with epilepsy should be referenced to the findings of semiology, EEG, and other neuroimaging methods.

Traditionally, the hippocampus is manually segmented on serial sections of a T1-weighted MRI scan. Although manual segmentation by experts in neuroanatomy has been the accepted standard, this approach is time consuming and requires a trained operator with a reliable and consistent rating method to maintain low interrater variability. A number of software-based approaches have been developed to segment the hippocampus in MRI without manual intervention (21, 22). Derived from these automated approaches, various computational techniques have been used to measure changes in the hippocampal volume in patients with Alzheimer's disease, Parkinson's disease and MTLE (23–25). Based on converging data obtained from the shape and volume measures of the hippocampus in healthy volunteers, Morey et al. concluded that FreeSurfer was generally preferred to FSL-FIRST for automated hippocampal segmentation (26). Pardoe et al. compared two automated software-based (FreeSurfer and FSL-FIRST) hippocampal volume methodologies and manual hippocampal volumetry in patients with MTLE; the authors concluded that FreeSurfer was more sensitive at detecting hippocampal atrophy and could be used if an expert in manual segmentation is unavailable (25). Based on the abovementioned research on healthy volunteers and patients with MTLE, we used FreeSurfer to perform automated segmentation of the hippocampus in the present study.

We used 3D T1-weighted MRI and FDG-PET images for the quantitative analyses, which are already the routine imaging modalities for patients with epilepsy and do not require any additional neuroimaging scans. With the exception of the commercial MATLAB® platform, all the software (SPM8, FreeSurfer and the Error ellipse script) required for our method is freely available. A script was written to automatically run the quantitative analysis without any manual intervention. During testing on a 12 Core CentOS 6.4 machine with 48 GB of memory, all processing was completed within ~6–8 h. Brain segmentation by FreeSurfer requires the major block of time in this workflow. The main limitation of the present study is small sample size, and prospective, multicenter, larger cohort studies are needed to test the performance of the methods we proposed.

## Conclusions

In MRI or ^18^FDG-PET images that are visually assessed by experts, the quantification of hippocampal volume or glucose uptake can improve the detection of HS in patients with MTLE. The two quantitative methods are objective, easily available, time efficient, economic, and appear to be valuable additional diagnostic tools in the evaluation of patients with epilepsy who are suspected having of HS.

## Ethics statement

Written informed consent was obtained from all included subjects, and protocols were approved by the Institutional Review Boards of the Beijing Tiantan Hospital. The study was performed in accordance with the ethical standards of the 1964 Declaration of Helsinki and its later amendments.

## Author contributions

WH: acquisition of data, statistical analysis, and drafting the manuscript; LL, BZ, XW, and CZ: acquisition and interpretation of data, revising the manuscript for intellectual content; XS, KZ, Y-SM, and LA: acquisition of data and revising the manuscript for intellectual content; JL: acquisition and interpretation of data; JZ: study design, study supervision, and final revising the manuscript for intellectual content.

### Conflict of interest statement

The authors declare that the research was conducted in the absence of any commercial or financial relationships that could be construed as a potential conflict of interest.
